# Supplementary material for: MCAs in Arabidopsis are Ca2+-permeable mechanosensitive channels inherently sensitive to membrane tension
Source: Nat Commun. 2021 Oct 19;12:6074. doi: 10.1038/s41467-021-26363-z (PMC8526687; doi:10.1038/s41467-021-26363-z)
Supplement: Supplementary file 1 — Supplementary Information [file 41467_2021_26363_MOESM1_ESM.pdf]

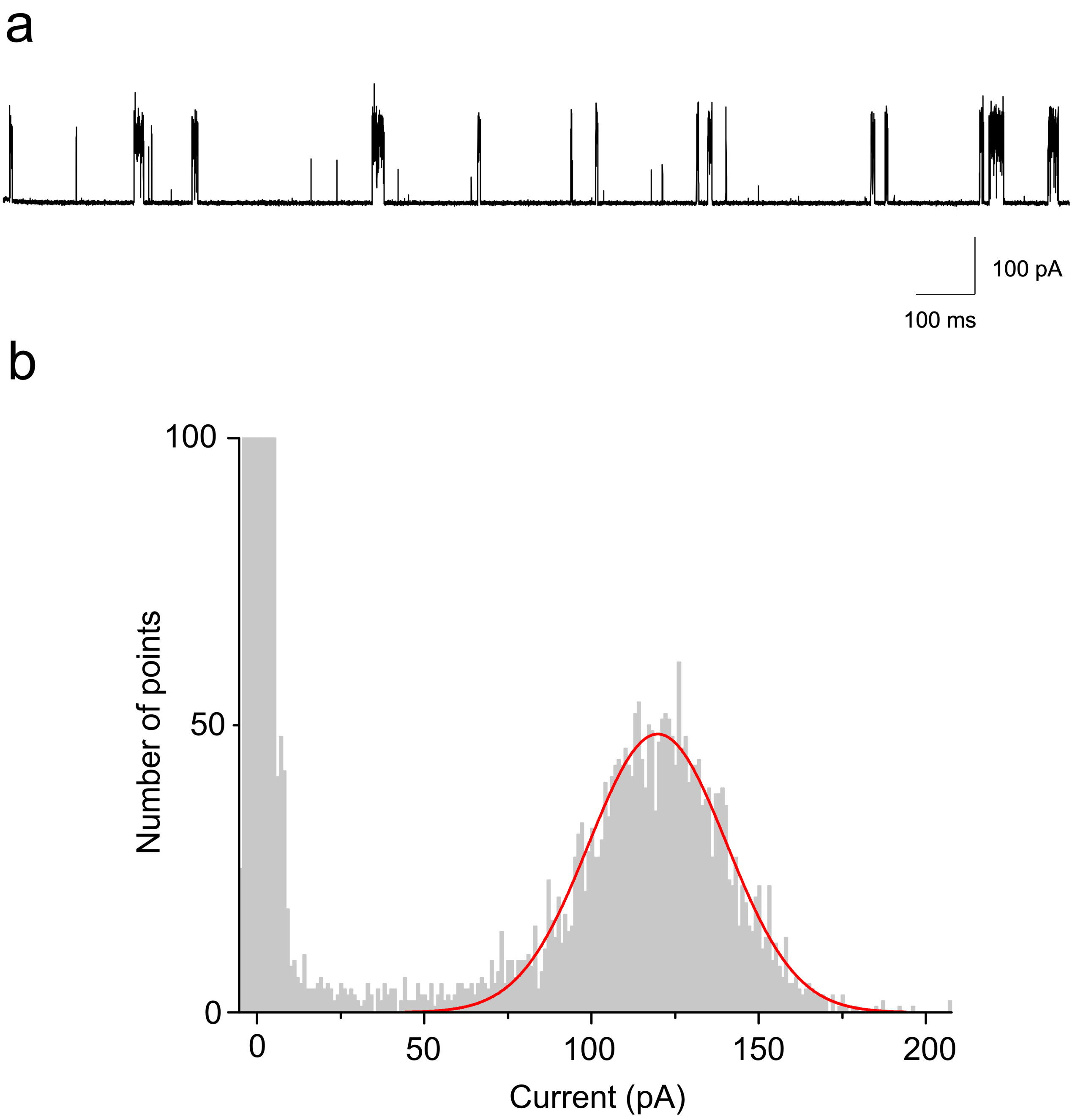

Supplementary Figure 1. All point histogram of a current trace.
(**a**) Current recording obtained at +200 mV. This trace includes the episode shown in Fig. 4a. (**b**) All point histogram of the trace shown in panel (**a**). The vertical axis is expanded to show the peak of the open state; therefore, the peak of the closed state is out of range in the graph. The distribution of the open state is fit with a single gaussian distribution, as shown by a red curve.
